# NUT midline carcinoma as a primary lung tumor treated with anlotinib combined with palliative radiotherapy: a case report

**DOI:** 10.1186/s13000-021-01188-y

**Published:** 2022-01-07

**Authors:** Jin Jiang, Yikun Ren, Chengping Xu, Xing Lin

**Affiliations:** 1 Department of Neurology, The First Affiliated Hospital of Chongqing Medical University, Chongqing, 400016 China; 2 Department of Pathology, The Southwest Hospital, the Southwest Hospital of Army Medical University, Chongqing, 400038 China; 3 Department of Biological Immunotherapy, Chongqing University Cancer Hospital, Chongqing Cancer Institute, Chongqing Cancer Hospital, Shapingba District, Chongqing, 400030 China

**Keywords:** NUT midline carcinoma, Primary lung tumor, Anlotinib, Palliative radiotherapy, Case report

## Abstract

**Background:**

NUT (nuclear protein in testis) midline carcinoma (NMC) is a rapidly progressive tumor arising from midline structures. Recent cases have reported that the poor prognosis with a median survival of 6.7 months and a 2 years overall survival of 19% due to limited treatment. Based on the effect of arotinib on inhibiting tumor growth and angiogenesis. We present one patient case treated with anlotinib and radiotherapy.

**Case presentation:**

Here, we describe a 33-year old patient who complained of cough and chest pain and was diagnosed as a pulmonary NMC through CT scan, FISH and immunohistochemistry. In addition, we initially demonstrated that anlotinib combined with palliative radiotherapy could significantly prevent the tumor growth in a pulmonary NMC.

**Conclusion:**

The report indicated that anlotinib combined with palliative radiotherapy could inhibit the tumor progression in a pulmonary NMC, which may provide a combined therapy to pulmonary NMC in the future.

## Background

NUT midline carcinoma (NMC) is a rapidly progressive tumor arising from midline structures, including upper aerodigestive tract and mediastinum [1]. Less than one hundred cases have been reported worldwide until now. It is still conventional for its treatments, such as chemotherapy, radiotherapy and targeted therapy [2]. Recent cases have reported that the poor prognosis with a median survival of 6.7 months and a 2 years overall survival of 19% [3–5].

To be our knowledgement, angiogenesis plays an important role in tumor growth. Recent studies indicated that blocking angiogenesis has been a successfully alternative strategy in the managemenyt of cancer [4]. Stikingly, anlotinib has inhibitory effects on tumor progression via preventing angiogenesis. Its mechanism is about a novel receptor tyrosine kinases (RTK) inhibitor targeting vascular endothelial growth factor receptor (VEGFR)-2 and − 3, fibroblast growth factor receptor (FGFR) 1–4, platelet-derived growth factor receptor (PDGFR) -α and -β, c-Kit and Ret [5]. Of note, anti-angiogenesis drug has become an essential therapy for non small-cell lung cancer (NSCLC) [5]. A pulmonary NMC characterized by a highly aggressive cancer of squamous cell carcinoma.

Therefore, in this report, we utilized radiotherapy and anlotinib to inhibit tumor growth, which may show an novel option to treat a pulmonary NMC.

## Case presentation

**Fig. 1:**
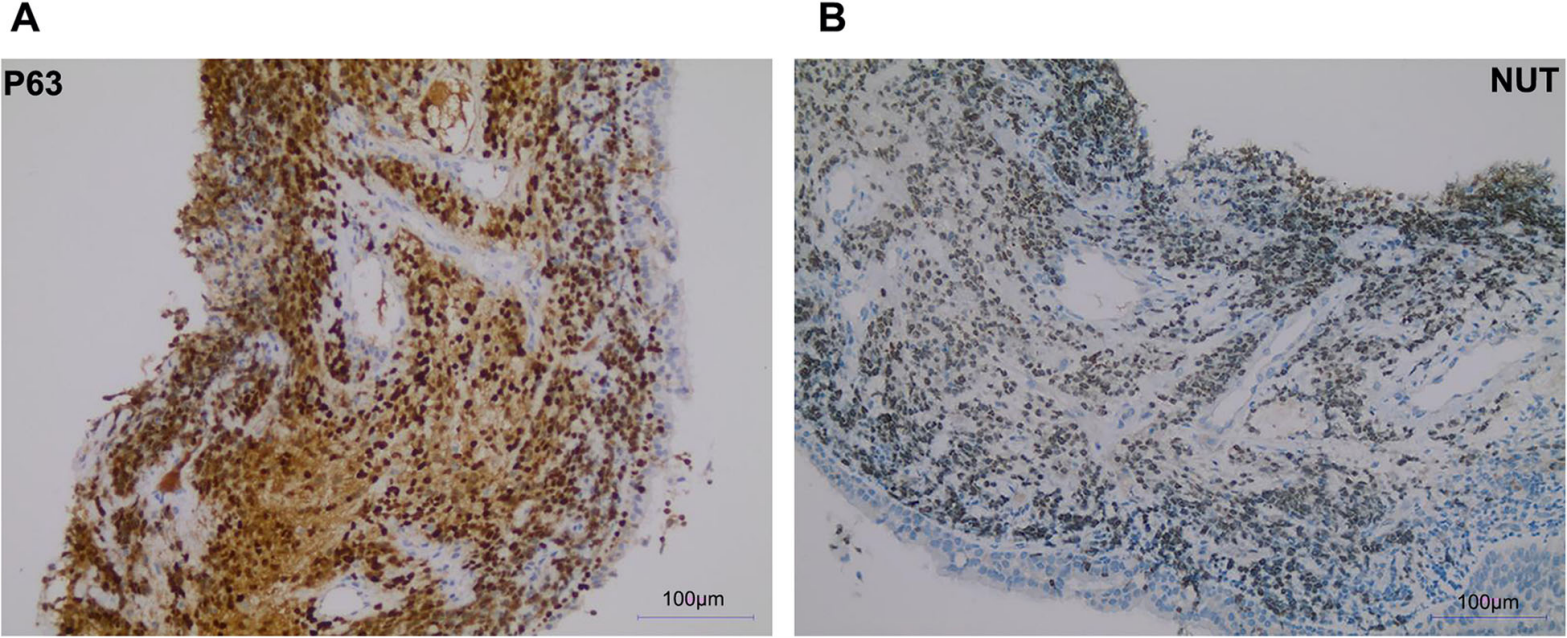
IHC staing showed markedly positive. **A:** P63 was the positive one which supported squamous cell carcinoma. **B:** IHC staing with NUT protein showed obviously positive. Scale bar = 100 μm

**Fig. 2:**
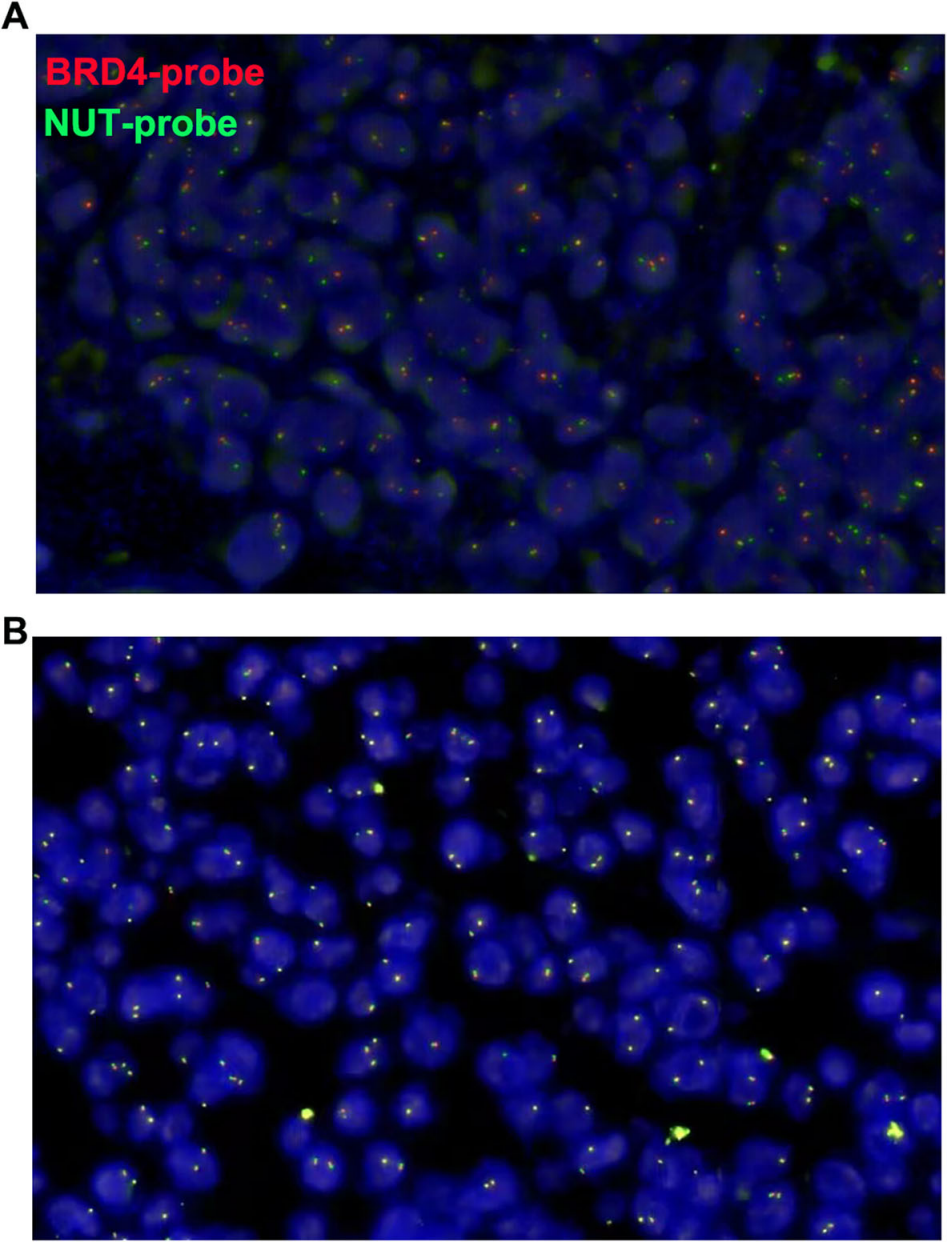
**A**: Fluorescent DNA in situ hybridization (FISH) demonstrated a BRD4-NUT rearrangement. **A**: A red probe that spans NUT splits and joins the green BRD4 centromeric probe. **B**: A negative control. Scale bar = 50 μm

**Fig. 3:**
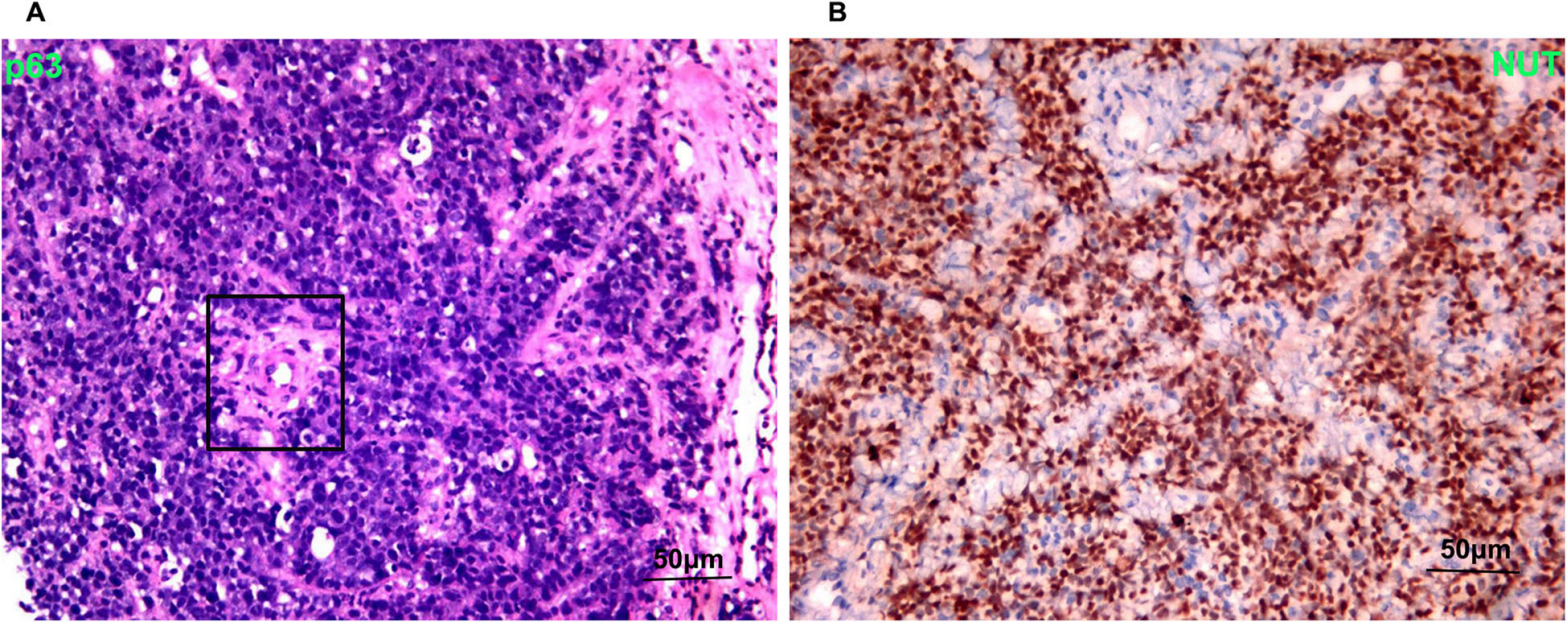
NUT midline carcinoma as a primary lung tumor. **A**: The NMC CT on Sept. 9th 2020. **B**: The NMC CT on Sept. 25th 2020. **C**: The NMC CT on Nov. 9th 2020

**Fig. 4:**
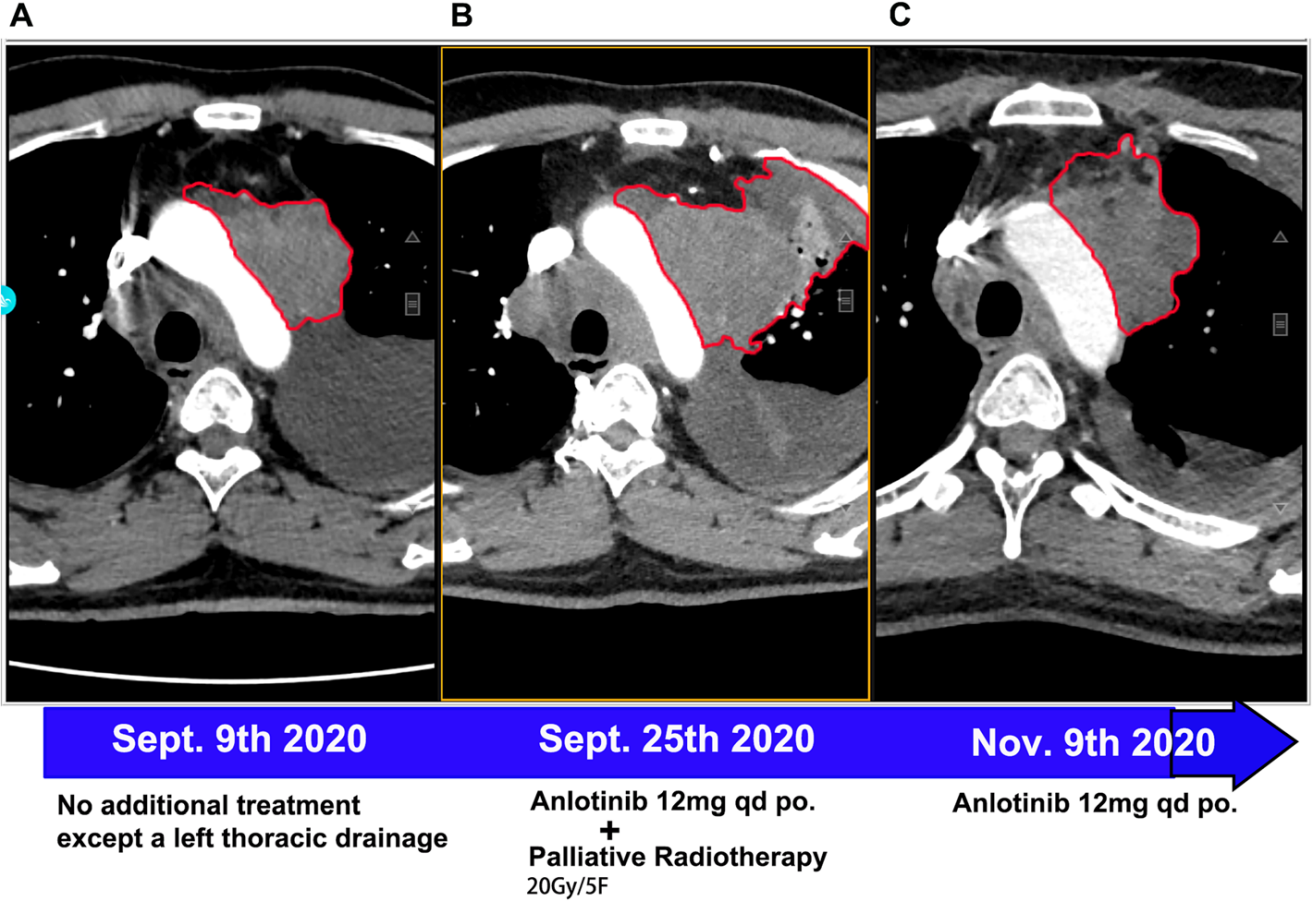
The biopsy tissue from left supraclavicular lymph node showed that the possible metastases from pulmonary NMC. **A**: Hematoxylin-eosin (HE) staining demonstrated a NUT midline carcinoma with aberrant squamous differentiation. **B**: IHC staining with p63 protein showed obviously positive. Scale bar = 50 μm

A 33 years old man with a smoking history of 18 pack-years complained of cough and chest pain for 1 month. He went to Southwest Hospital of Third Military Medical University. Transbronchial biopsy was routinely performed. A highly aggressive differential carcinoma considered as NMC was found in biopsy tissue. The immunohistochemical (IHC) markers revealed that p63(+) and NUT(+). P63 was the positive one which supported squamous cell carcinoma (Fig. 1A). IHC staining with NUT protein showed markedly positive (Fig. 1B). Fluorescent DNA in situ hybridization (FISH) test showed a BRD4-NUT rearrangement (Fig. 2A). NMCs are difficult to morphologically different from other poorly differentiated carcinomas, and the diagnosis is usually made currently by FISH. Herbert et al. Have reported that FISH for NUT rearrangement was used as a “gold standard” diagnostic test for NMC. C52 immunoreactivity among carcinomas was confined to NMCs. IHC staining had a sensitivity of 87%, a specificity of 100%, a negative predictive value of 99%, and a positive predictive value of 100% [6]. The diagnosis was confirmed as a pulmonary NMC via IHC since the anti-NUT C52 monoclonal antibody had a specificity of 100%. A chemotherapy (paclitaxel liposome 300 mg day1 and cisplatin 120 mg day1). One month later, he came to our hospital because of serious shortness of breath. Physical examination showed that the breathing of the left lung was low. Hematoxylin-eosin (HE) staining demonstrated a NUT midline carcinoma with aberrant squamous differentiation (Fig. 3A). IHC staining with p63 protein showed obviously positive (Fig. 3B). Chest CT revealed that a perihilar soft tissue mass which near to hilar and mediastinal part measuring 5.6 cm*5.1 cm in the left lower lober lung, left upper lobe obstructive inflammation, possible metastases of nodules and masses in the lower lobe of the left lung, possible metastases of left supraclavicular, left chest wall and axillary lymph nodes and superiorvena cava and left brachiocephalic vein compression and left pleural effusion (Fig. 4A). Abdominal CT showed that neither heptic nor adrenal metastases. ^99m^Tc bone scan and brain magnetic resonance imaging (MRI) demonstrated that no evidence of bone and brain metastases. The biopsy tissue from left supraclavicular lymph node showed that the possible metastases from pulmonary NMC. No additional treatment except a left thoracic drainage such as chemotherapy or radiotherapy was added. Twenty-one days later, he came back to our hospital since the shortness of breath became more serious. Physical examination showed that serious facial edema. Chest CT revealed that a perihilar soft tissue mass which became larger measuring 9.7 cm*9.0 cm in the left lower lober lung (Fig. 4B). Emergency palliative radiotherapy (20Gy/5F) to malignant tumor of the left lower lober lung for alleviating superior vena caval syndrome (SVCS) and anlotinib (12 mg qd po.) for antiangiogenesis were administrated. Sixty days later, CT scan showed that the perihilar soft tissue mass which became absolutely smaller measuring 5.0 cm*3.7 cm in the left lower lober lung (Fig. 4C). However, palliative radiotherapy and targeted therapy were not subsequently administrated because of radiation esophagitis, radiation stomatitis and bone marrow suppression after ten days later. He passed away 4 months after diagnosis.

## Discussion and conclusions

NMC characterized by chromosomal rearrangement of the involvement of nuclear protein in testis (NUT) of the testis on 15q14 is a highly aggressive cancer of squamous cell carcinoma, which always arising in the midline structure of the body, such as head, neck, the upper aerodigestive tract and mediastinum [7]. The specific molecular genetics is that the fusion oncogene BRD4-NUT as result from BRD4 gene translocation located at 19p13.1 in the NMC [8]. Therefore, it was initially classified carcinoma with t (15;19) by WHO Classification of Tumours in 2004 [8]. Previous studies have shown it to be associated with Epstein Barr virus (EBV) and Human Papilloma virus (HPV) [9]. Kees and Kubonishi have reported the first two cases which were considered arising from thymus in 1991 [10]. As the fusion genes discovered by FISH assay, NUT carcinoma was newly classified by WHO in 2015 [6].

Previous studies have demonstrated that the poor prognosis in NMC cases with a median survival at less than approximate 9 months [11]. To be our best knowledge, its incidence and underlying mechanism were not clear. According to previous reports, patients of all age groups ranged from several months to 78 years old, especially children and young adults [12]. The most common clinical symptom was cough [13]. However, cough often had not been noticed during the early stage. The lung is one of the most common sites for NUT cancer, but there are few reports on lung NUT cancer. The largest centralized report is a retrospective analysis published in the Journal of Thoracic Cancer in 2015 [14], a review of the study The clinical features of 8 cases of lung NUT cancer were analyzed. Unfortunately, the average survival time of 8 patients was only 2.2 months, which was much lower than the 5.5-month average survival time of 119 cases of NUT cancer reported by Giridha [15]. It was unfortunate that most patients had lose the surgery chance because of its distant metastases. Even so, there is no standard treatment to NMC. We noted that the majority of patients were administrated chemotherapy and/or radiotherapy [15]. Here, for the first time we revealed that the primary pulmonary NMC, initially treated with the combination of radiotherapy and anlotinib for antiangiogenesis.

It has been acknowledged that anlotinib plays inhibitory effects on tumor growth and angiogenesis because anlotinib is a novel RTK inhibitor targeting VEGFR-2 and -3, FGFR 1–4, PDGFR-α and-β, c-Kit and Ret [16]. Arotinib have received its first approval as a third-line treatment for refractory advanced NSCLC in May 2018 [17]. Recent studies have demonstrated that anlotinib increases the sensitivity of pulmonary blastoma to chemotherapy in vivo [18]. As we know, activation of the FGFR signaling pathway promotes chemotherapy resistance. Interestingly, anlotinib could inhibit the tumor growth by suppressing the activation of FGFR1–4 [18]. Thus, we speculated that anlotinib may exert a positive role on overcoming the chemotherapy resistance. Shi et al. have suggested that combination therapy with anlotinib and chemoradiotherapy may be an effective regimen for the treatment of advanced esophageal squamous cell carcinoma (ESCC) [19]. A study by Wang et al. indicated that stereotactic radiosurgery (SRS) combined with anlotinib limited brain metastases with perilesional edema in NSCLC [20]. A recent study has reported that radiotherapy combined with anlotinib to treat with orbital NMC [21]. In this report, we for the first time used palliative radiotherapy combined with anlotinib could significantly alleviate primary lesion of NMC progression, which may provide a way to treat primary pulmonary NMC. We speculate that anlotinib may exert a positive role on improving the radiotherapy sensibility, which needs our advanced investigation in the future.

In summary, we obtain the valuable experience to realize the characteristic of NMC in this present case. Moreover, we initially reported a case of primary pulmonary NMC, which was suppressed the progression by radiotherapy combination with anlotinib. If NMC is diagnosed at the early stage, surgery may be more effective. However, radiotherapy may play an important role in the process of treatment during the advanced stage of NMC. Additionally, anlotinib could be considered to be the treatment of primary pulmonary NMC. Further studies and clinic case reports are essential to explore underlying mechanisms in NMC.

## Data Availability

N. A.

## Abbreviations

NMC: nuclear protein in testis midline carcinoma
RTK: receptor tyrosine kinases
VEGFR: vascular endothelial growth factor receptor
FGFR: fibroblast growth factor receptor
PDGFR: platelet-derived growth factor receptor
NSCLC: non small-cell lung cancer
CT: computed tomography
FISH: fluorescent DNA in situ hybridization
MRI: magnetic resonance imaging
SVCS: superior vena caval syndrome
EBV: Epstein Barr virus
HPV: human papilloma virus
ESCC: esophageal squamous cell carcinoma
SRS: stereotactic radiosurgery
